# A Model for the Gene Regulatory Network Along the Arabidopsis Fruit Medio-Lateral Axis: Rewiring the Pod Shatter Process

**DOI:** 10.3390/plants13202927

**Published:** 2024-10-18

**Authors:** José Moya-Cuevas, Elizabeth Ortiz-Gutiérrez, Patricio López-Sánchez, Miguel Simón-Moya, Patricia Ballester, Elena R. Álvarez-Buylla, Cristina Ferrándiz

**Affiliations:** 1 Instituto de Biología Molecular y Celular de Plantas, Consejo Superior de Investigaciones Científicas–Universidad Politécnica de Valencia, 46022 Valencia, Spain; jose.moya@uma.es (J.M.-C.); miguelsimonmoya@gmail.com (M.S.-M.); pballester@ibmcp.upv.es (P.B.); 2 Instituto de Hortofruticultura Subtropical y Mediterránea, Universidad de Málaga-Consejo Superior de Investigaciones Científicas (IHSM-UMA-CSIC), Campus de Teatinos, 29071 Málaga, Spain; 3 Departamento de Ciencias Naturales, Unidad Cuajimalpa, Universidad Autónoma Metropolitana, Mexico City 05348, Mexico; elizabeth.ortiz@conahcyt.mx (E.O.-G.); patrick.losa90@gmail.com (P.L.-S.); 4 Laboratorio de Genética Molecular, Epigenética, Desarrollo y Evolución de Plantas, Instituto de Ecología, Universidad Nacional Autónoma de México, Mexico City 04510, Mexico; eabuylla@gmail.com

**Keywords:** fruit, dehiscence, systems biology, Boolean modeling, GRN, Arabidopsis

## Abstract

Different convergent evolutionary strategies adopted by angiosperm fruits lead to diverse functional seed dispersal units. Dry dehiscent fruits are a common type of fruit, characterized by their lack of fleshy pericarp and the release of seeds at maturity through openings (dehiscence zones, DZs) in their structure. In previous decades, a set of core players in DZ formation have been intensively characterized in Arabidopsis and integrated in a gene regulatory network (GRN) that explains the morphogenesis of these tissues. In this work, we compile all the experimental data available to date to build a discrete Boolean model as a mechanistic approach to validate the network and, if needed, to identify missing components of the GRN and/or propose new hypothetical regulatory interactions, but also to provide a new formal framework to feed further work in Brassicaceae fruit development and the evolution of seed dispersal mechanisms. Hence, by means of exhaustive in-silico validations and experimental evidence, we are able to incorporate both the NO TRANSMITTING TRACT (NTT) transcription factor as a new additional node, and a new set of regulatory hypothetical rules to uncover the dynamics of Arabidopsis DZ specification.

## 1. Introduction

**Figure 1:**
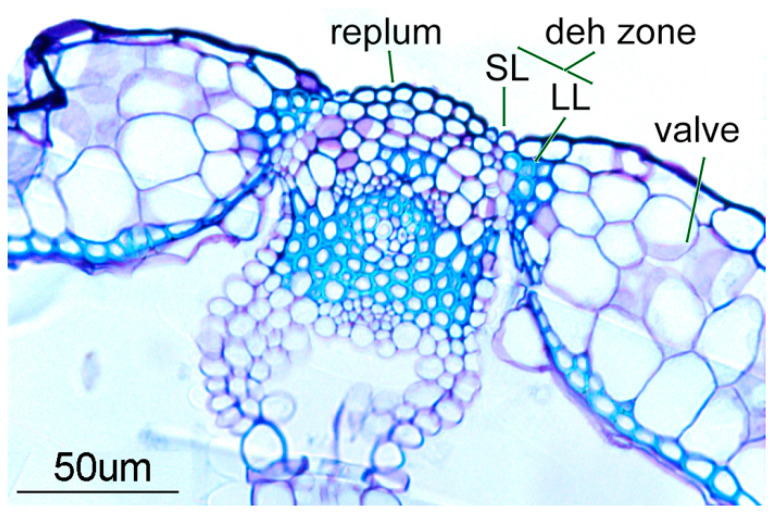
The transversal section of an Arabidopsis mature silique showing the essential tissues required for efficient seed dispersal. SL: separation layer; LL: lignification layer; deh zone: dehiscence zone.

Fruit is a key evolutionary innovation of flowering plants, responsible for the protection and dispersal of developing seeds. Fruits can be divided in two categories: dry and fleshy. While the latter have evolved to be attractive to animals that eat them and thus act as vectors for seed dissemination, dry fruits usually rely on wind or other mechanical forces to disperse seeds. Many dry fruits open at maturity to release seeds directly into the environment. For this, they must undergo the so-called dehiscence or pod shatter process, which usually involves the development of specialized tissues that ultimately allow the controlled opening of the fruit at the optimal stage of seed maturation [1]. The mode of fruit aperture is an important ecological and agronomic trait for crop improvement, and hence, over the last two decades, many research efforts have focused on understanding the molecular basis of the dehiscence process mainly in the model plant *Arabidopsis thaliana*. In the ovary of Arabidopsis pods, three different zones can be distinguished: the valves, the replum, and the valve margins (Figure 1). The pod walls (the valves) ensure the protection and development of the seeds until their optimum maturity, at which point they detach, promoting seed dispersal. The two valves are separated by the replum, a narrow domain external to the main vascular bundles, corresponding to the outer part of the septum that divides the ovary longitudinally. The valve margin tissues are placed at the valve/replum junction to allow for the detachment of both valves from the replum at pod maturity. The valve margin comprises two specialized cell types. On the one hand, the separation layer (SL) is formed by small cells adjacent to the replum side, where it facilitates valve detachment through cell–cell separation processes [2,3]. On the other hand, a cell stripe of lignified cells (the lignification layer, LL) comes up on the valve side of the margin to likely provide, together with the SL, the required spring-like forces that mechanically trigger valve detachment [4].

**Figure 2:**
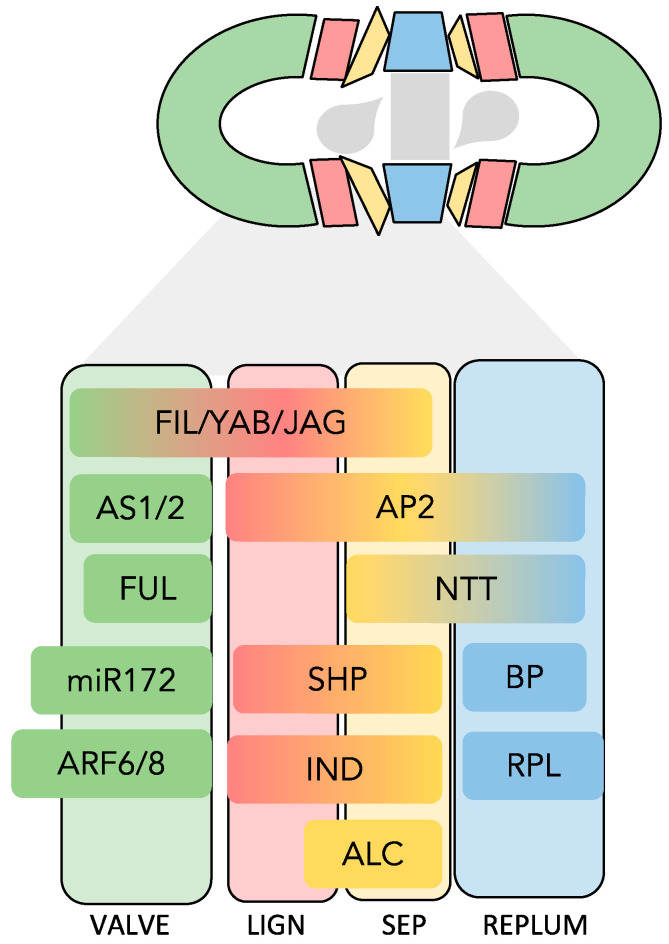
A schematic transversal section of an Arabidopsis mature silique focused on the expression domain(s) of the set of transcriptional regulators driving the specification of the dehiscence zone. Each color represents a functionally different tissue. LIGN: lignification layer; SEP: separation layer.

The key components of the gene regulatory network (GRN) driving the morphogenesis of the dehiscence zone (DZ) in Arabidopsis fruit have been known for quite some time. The core of this network can be ascribed only to the concerted action of relatively few transcription factors (TFs) (Figure 2). The functionally redundant MADS-box genes *SHATTERPROOF 1* (*SHP1*) and *SHP2* are expressed in the valve margin of the gynoecium and in young fruit, where they upregulate the basic helix–loop–helix (bHLH) genes *INDEHISCENT* (*IND*) and *ALCATRAZ* (*ALC*).

The impaired function of *SHP* or *IND* results in entirely indehiscent fruits with the absence of both separation (SL) and lignification (LL) layers, whereas *alc* mutants are only deficient in SL formation [5,6,7]. In addition, two additional regulators act as repressors in the valves and replum, respectively, *FRUITFULL* (*FUL*), another MADS-box gene, and the homeobox gene *REPLUMLESS* (*RPL*). FUL and RPL restrict the expression of the SHP/IND/ALC module to the valve margin domain, completing the basic GRN that substantially explains the emergence of the different cell types characterizing DZ formation [6,8,9]. However, this simplified scenario becomes increasingly complex as we incorporate additional modulators identified in more recent works, which are not essential for DZ specification but modify the extent and positioning of this domain in a partially redundant manner. Thus, the bHLH TF gene *SPATULA* (SPT), a close paralog of *ALC*, can partially substitute for *ALC* in SL formation [10], and it has also been shown that IND and SPT factors need to heterodimerize to regulate auxin dynamics in the fruit for proper SL formation [11]. The replum width is determined by additional factors, related to meristem functioning, that also act at the medial domain of the gynoecium, like BREVIPEDICELLUS (BP) [12]. The development of the two lateral pattern elements, the valve and valve margin, is directed by the synergistic activity of the genes *JAGGED* (*JAG*), *FILAMENTOUS FLOWER (FIL),* and *YABBY3* (*YAB3*), initially characterized by their role in leaf development [13]. Accordingly, several authors have proposed reciprocal antagonistic activities among medial (*BP/RPL*) and lateral factors (*JAG/FIL*) in the gynoecium, mimicking the relationship between genes maintaining the undifferentiated state of meristem and genes promoting the differentiation of leaves. In this same context, the *ASYMMETRIC LEAVES 1 (AS1)* and *AS2* genes are expressed in lateral domains and, when mutated, cause a significant reduction in valve width and concomitant replum expansion [14,15,16,17]. Another of these recently uncovered newcomers is *APETALA2 (AP2)*, better known as one of the homeotic genes that specify perianth organ identity, which in the gynoecium fine-tunes the expression of both DZ (*SHP/IND*) and replum (*RPL/BP*) factors to correctly delimit the size of these territories [18]. To conclude this overview of the experimentally well-supported participants that build the elementary scaffold of this medio-lateral network, it is mandatory to consider post-transcriptional regulation as well as the role of hormones. Thereby, the combined action of FRUITFULL (FUL) along with AUXIN RESPONSE FACTOR6 (ARF6) and ARF8 activates *miR172*, thus preventing ectopic AP2 activity in the valves, which results in fruit phenotypes that mildly resemble those of *ful* mutants [19]. On the other hand, by directly regulating a discrete number of downstream targets, such as the gibberellin (GA) biosynthetic enzyme GA3ox1, IND promotes the establishment of opposite local hormone gradients, where minimum auxin and cytokinin levels versus a gibberellic acid maximum at the valve/replum boundary act to instruct proper DZ development and pod shattering [11,20,21,22,23]. In this manner, the separation layer differentiates as a result of this increment in GAs at the DZ domain, where IND becomes an indirect activator of *ALC* through the degradation of DELLA repressor proteins, which in turn feed-back negatively on *IND* expression levels to prevent consequent IND-promoted lignification [20,24]. However, despite the exhaustive experimental data generated thus far, the complex dynamics underlying this network are not fully understood. For instance, it is unclear how *SHP* and *IND* drive the differentiation of lignified and separation layers in neighboring cell stripes, excluding *ALC* from the lignification layer to keep it confined only to the separation layer, considering that no repressors of *ALC* have been identified to date. In this study, we sought to use modeling tools to integrate the existing information into a minimal network comprising the set of necessary and sufficient components and regulatory interactions that shape the *A. thaliana* DZ to help to solve these inconsistencies and gaps in our knowledge.

We chose to use synchronous Boolean dynamic networks as a mechanistic approach to provide a systemic and formal working framework, by implementing a strategy for network inference that has been successfully deployed in different organisms and biological processes [25,26]. We found that, despite integrating all the previously published meaningful data related to DZ formation into a discrete Boolean model, the regulatory interactions previously published were not sufficient to explain the emergence of the expression patterns which conform to the four different resultant cell fates. Therefore, our results drew attention to the need to propose new hypothetical interactions and/or components, as well as to carry out more experiments or revisit the recent literature to incorporate additional elements to the network. We took these actions to expand the set initially considered, and then we subjected our proposed extended network to exhaustive validation tests (loss- and gain-of-function simulation lines, perturbations in the Boolean functions, and conversion to a continuous approximation model). In this way, we largely recovered the expected dynamic behavior of the DZ participants. Hereby, we presented an integrative model to formally tackle the mechanisms of DZ specification in Arabidopsis that could inspire future experimental and modeling studies to better understand the pod shatter process.

## 2. Results and Discussion

### 2.1. Compiled Regulatory Interactions Among Transcriptional Regulators Are Not Sufficient to Recover DZ Cell Type Activity Profiles

**Figure 3:**
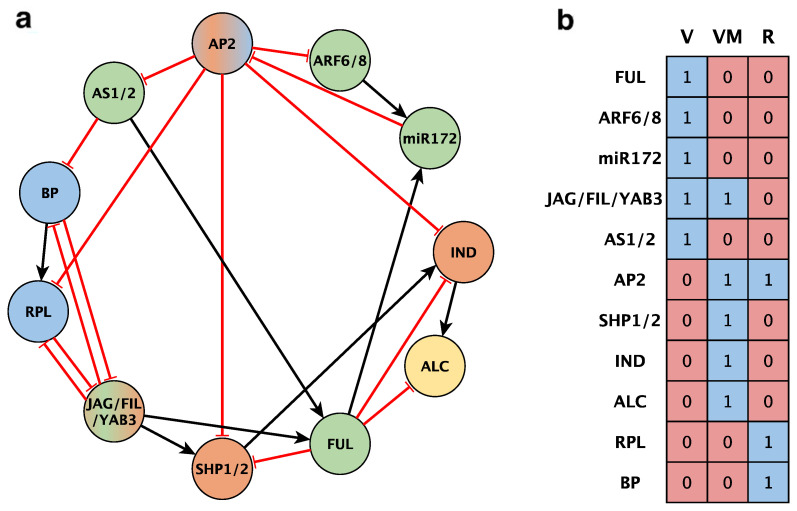
(**a**) A GRN proposed for the *A. thaliana* fruit dehiscence mechanism based on the literature. The network topology depicts the nodes considered in the model as well as the experimentally supported genetic interactions among them. Black edges with arrowheads are activating regulations and red edges with flat ends represent repressive regulations. Each node color represents a functionally different tissue. A dual color fill is for those active in more than one tissue. Green: valve (V); orange: valve margin (VM); yellow: separation layer (SL); blue: replum (R). (**b**) Attractors obtained with the network configuration in (**a**). Each column is the attractor that corresponds to a cell type, valve (V), valve margin (VM), or replum (R). Each network gene is represented by a table row. Red or 0 stands for a transcriptionally repressed gene or an absent protein; blue or 1 is for a transcriptionally active gene or a present protein.

Based on recent publications that proposed models for GRNs directing DZ formation in Arabidopsis, we aimed to build a minimal set of nodes corresponding to the genetic factors that were well characterized at the functional level, and for which detailed experimental evidence describing expression patterns and molecular interactions was available in wildtype and mutant backgrounds. After an extensive literature review, the resulting set of nodes and interactions was compiled as it appears in Table 1 and graphically described in Figure 3a. In addition, according to published patterns of expression or defined domains of activity, we also generated a combination of expression profiles for the four functional tissues at the medio-lateral plane of the fruit: the valve (V), lignified layer (LL), separation layer (SL), and replum (R). Our starting components were then a set of 11 nodes and 22 experimentally validated interactions. We then described the logical rules derived from these nodes/interactions to generate a Boolean model that was expected to recover the four functional domains required for DZ formation (Table S1) in the form of stable attractors. However, after running the corresponding scripts in the *Boolnet R* package [27], we were only able to obtain the configuration shown in Figure 3b, which lacks the proper differentiation of lignification and separation layers present in the Arabidopsis dehiscent fruit, and thus was dysfunctional. These results indicated that the set of experimental data that we exhaustively compiled was insufficient to explain the genetic mechanisms driving the differentiation of the DZ. As a second attempt to infer a set of meaningful Boolean networks that were coherent with our input interactions, we used the *Griffin* tool [28] using additional biological constraints, such as our set of expected fixed-point attractors for both the wild-type and mutant phenotypes. However, no meaningful networks were obtained following this additional approach, probably because the available information was insufficient (see queries for *Griffin* in the Methods section).

### 2.2. A New Node Added to the DZ GRN: NO TRANSMITTING TRACT (NTT) Factor

In the light of the results obtained, we considered the possibility of including new/alternative interactions and/or even additional nodes not necessarily derived from the experimental data exhaustively validated, and/or proposed based on indirect observations or interpretations of phenotypic effects of mutant combinations.

**Figure 4:**
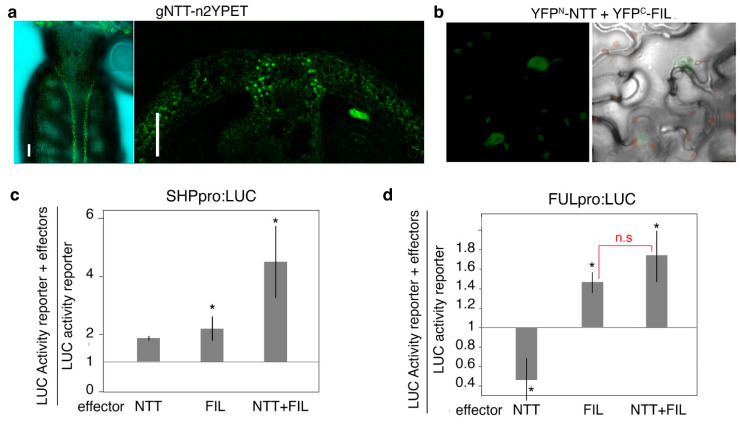
(**a**) The localization of NTT-n2YPET in anthesis ovaries. The signal is clearly detected along a narrow stripe in the valve margin (**left**), that in a transversal section appears to be confined to the separation layer. (**b**) A BiFC assay showing fluorescence complementation mediated by the interaction of NTT and FIL. Controls for the BiFC experiment are shown in Supplementary File S4. (**c**,**d**) Transient assays of SHPpro:LUC (**c**) and FULpro:LUC (**d**) expression. SHP:LUC-35S::REN and FUL:LUC-35S::REN reporter constructs were transiently expressed in *Nicotiana benthamiana* leaves either alone or together with effectors 35S:NTT, 35S:FIL, or both. The expression of REN was used as an internal control. LUC activity was normalized with REN in each case and the relative activity of the reporter + effectors to the reporter alone was calculated (n = 6). Asterisks indicate significant differences according to a Student’s *t*-test (*p* < 0.05) from the values obtained when the promoter::LUC-35S::REN reporters were infiltrated alone, while n.s. means no significant differences between the indicated data.

The *NTT’s* role in fruit development has been described in different publications [43,44,48,49,50]. Interestingly, while *ntt* loss of function does not significantly impact the development of the DZ, replum, or valves, *NTT* overexpression causes a major perturbation of the distribution and identity of these domains, with phenotypes resembling *ful* mutants, a concomitant reduction in *FUL* expression levels, and the ectopic expression of *BP* (see Table 1). However, *NTT* expression has not been robustly characterized, and several publications report conflicting expression patterns in the fruit, making it difficult to ascribe its activity to specific domains in the expected set of attractors [44,49,50]. To clarify this point, we made use of a pNTT:gNTT-n2YPET line [51] to carefully examine NTT protein localization, which was detected mainly in the SL (Figure 4a). Close inspection of the published characterization of the ntt mutant phenotypes in the fruit showed a slight shift of the SL to the LL in the ntt mutants [44], consistent with the role of *NTT* in the correct SL specification, and thus we decided to include *NTT* as a functional node in the SL. Additional published studies [49] also showed preferential expression of *NTT* in the replum and, since the positive regulation of *BP* (a replum factor) by NTT was well established, we also ascribed NTT as a functional node in the replum, despite our confocal images of mature siliques not showing clear expression in this domain.

FIL and JAG have been described as *FUL* and *SHP* positive regulators (Table 1) [13,14,38]. Since *FUL* and *SHP* are expressed in the valve and valve margin, respectively, it has been proposed that this differential activation of *FUL* and *SHP* in adjacent domains was mediated by a putative gradient of FIL/JAG concentration decreasing towards the valve margins. High levels of these factors could activate *FUL* and possibly *SHP*, which in turn would be repressed by FUL, while the reduced concentration of FIL/JAG in the valve margins would be sufficient to activate *SHP* but not *FUL* [15]. However, this hypothesis was based on genetic evidence, but it has not been conclusively proven. Because NTT has been shown to interact with FIL and JAG (Figure 4b) [48], and NTT overexpression phenotypes suggested NTT as a putative repressor of *FUL,* we explored an alternative scenario in which FIL/JAG activity on *FUL* and *SHP* promoters would be modulated by the presence of NTT to establish this differential output. The activity of *FUL* and *SHP* promoters was assayed in transient expression analyses in *N. benthamiana* leaves in response to different combinations of effectors (Figure 4c,d). When acting alone, NTT was a repressor of *FUL* promoter activity, but a weak activator of *SHP*, while FIL was able to induce the activity of both *FUL* and *SHP* promoters. Surprisingly, the FIL+NTT combination resulted in the enhanced activation of the *SHP* promoter activity and suppressed the negative effects of NTT on *FUL* promoter regulation (Figure 4c,d). If NTT is assumed to be excluded from the valves, these results could explain why *FUL* can be activated by FIL in this domain while repressed in the replum by NTT, while in the valve margin, when FIL/JAG and NTT overlap, *SHP* activation would be enhanced and overrule *FUL* activation. In the context of our current work, these results were also used to propose more logical rules, considering that JAG/FIL/YAB3 are positive activators of *FUL* only if NTT is not present and that JAG/FIL/YAB3 are positive activators of *SHP* if NTT is present.

### 2.3. The Proposed Network with Novel Predicted Regulatory Interactions and Nodes Recovers the Expected Configurations of the DZ Cells

**Figure 5:**
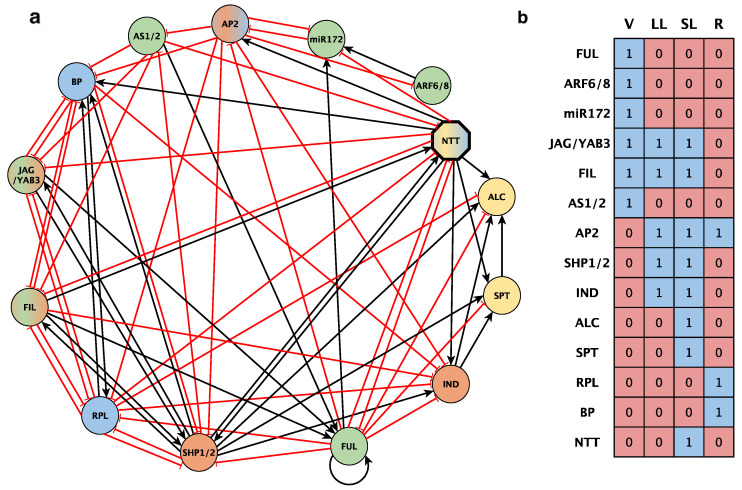
(**a**) A GRN proposed for the A. thaliana DZ including NTT and the set of novel interactions. Black edges with arrowheads are activating regulations and red edges with flat ends represent repressive regulations. Each node color represents a functionally different tissue. An octagonal bold line shape is for a novel NTT node. Dual color fill is for those active in more than one tissue. Green: valve (V); orange: valve margin (VM); yellow: separation layer (SL); blue: replum (R). (**b**) Recovered attractors corresponding to those expected for a dehiscent Arabidopsis fruit. Each column is the attractor that corresponds to a cell type, valve (V), lignification layer (LL), separation layer (SL), or replum (R). Each network gene is represented by a table row. Red or 0 stands for a transcriptionally repressed gene or absent protein; blue or 1 is for a transcriptionally active gene or a present protein.

The addition of NTT together with the novel proposed interactions (Figure 5a) was sufficient for Boolnet to recover the expected four attractors in the ovary (Figure 5b).

However, in addition to these expected fixed-point attractors, some cyclic attractors were also obtained which did not correspond to any experimentally observed state (Supplementary File S3). These cyclic attractors are not unusual outcomes of the Boolean model, where occasional artifactual outputs caused by the intrinsic characteristics of Boolean assumptions can be found when transitions between the different configurations of the network are less robust, making reversions possible. To distinguish this artifactual behavior of the model from a real cyclic attractor, it is possible to translate any regulatory network into a continuous dynamical system with the use of a set of ordinary differential equations (see Section 3). In the Supplementary File S3, we demonstrate how all the nodes in the network reach a stationary state eliminating the cyclic attractor, which corresponds instead to a stationary state equivalent to that of the SL.

It is also worth mentioning here that to build the model we used robustly validated interactions and a limited number of suggested/hypothetical interactions, but we also introduced a significant arbitrary set of logical operators (AND/OR/ONLY/NOT) within the logical rules operating among the nodes. While these have limited biological meaning at this point, they represent a valuable tool to test new hypotheses and to understand the complexity of the dynamic relationships of the network. Moreover, once the model is generated, it is possible to test in silico both these and/or new hypotheses to reduce the experimental work required to confirm our predictions and to uncover new key elements or interactions required for the correct development of the DZ.

### 2.4. The Recovered Configurations of the Newly Proposed DZ Model Are Robust to Perturbations

**Figure 6:**
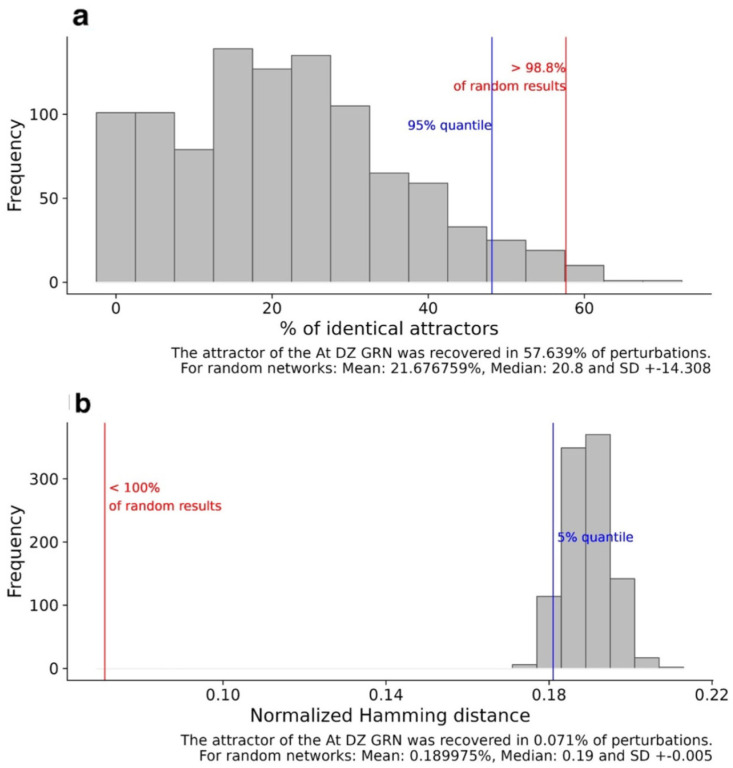
Attractor robustness analysis. The attractors recovered after perturbing either the Boolean functions (**a**) or transition states (**b**) of the DZ GRN and similar random networks. The red line represents the result corresponding to the DZ network model. The blue line determines the significance level calculated from inducing the same type of perturbations to similar random networks. In panel (**a**), the bars represent the frequency with which random networks recovered a certain percentage of their original attractors. Panel (**b**) illustrates the normalized Hamming distance between the successor states of the original and the perturbed network obtained after perturbing the state transitions in random networks and the dynamic GRN dehiscence model.

To additionally assess the optimization of our rewired GRN model, two robustness analyses were performed by inducing random alterations in the Boolean functions or in the transition states between the network configurations (see Section 3). In the first analysis, the Boolean functions were altered in the proposed DZ network and on 1000 random networks with a topology similar to that of the DZ GRN. As a result, the simulation of perturbed functions revealed that the newly DZ network model still recovered its original attractors in 57.64% of the altered Boolean functions, in contrast to the set of randomly generated networks, which recovered their original attractors with a median of 20.8% (Figure 6a).

In the second robustness analysis, the differences between the original and the perturbed transition states, measured by the Hamming distance, were 0.071 for this novel dehiscence network, much lower than the average distance of 0.190 ± 0.005 SD between the original and altered transition states of the randomly generated networks. In both cases, the results were statistically significant with a *p*-value < 0.05 (Figure 6b). This output evidences the higher robustness to perturbations when comparing the new DZ architecture with similar artificial networks, validating its usefulness for this particular biological process.

### 2.5. Loss- and Gain-Of-Function Mutant Simulations Mostly Recover the Experimentally Observed Attractors

**Figure 7:**
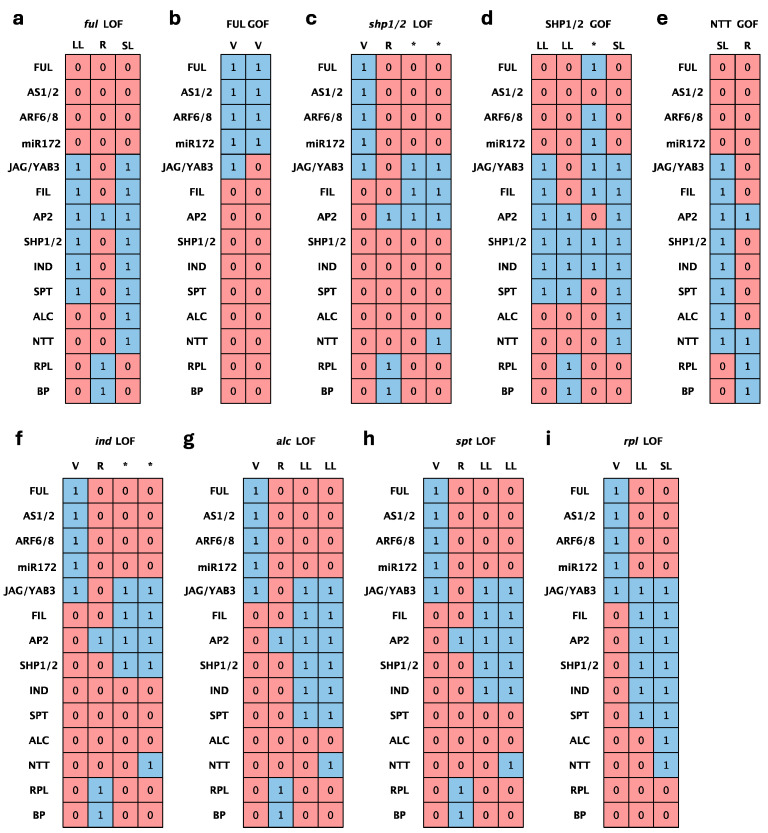
Attractors recovered by loss- or gain-of-function mutant simulations of selected Arabidopsis DZ regulators. Each column is an attractor configuration: valve (V), lignification layer (LL), separation layer (SL), or replum (R). The rows represent the state of each node: the squares in blue indicate nodes that are in an “ON” state and those in red are in an “OFF” state. Columns labeled with an asterisk indicate attractors with differences from the four canonical configurations. (**a**) Simulation of *FUL* loss-of-function (*ful* mutant). (**b**) Simulation of FUL gain-of-function. (**c**) Simulation of *SHP1* and *SHP2* loss-of-function (*shp1 shp2* double mutant). (**d**) Simulation of SHP1 and SHP2 gain-of-function. (**e**) Simulation of NTT gain-of-function. (**f**) Simulation of *IND* loss-of-function (*ind* mutant). (**g**) Simulation of *ALC* loss-of-function (*alc* mutant). (**h**) Simulation of *SPT* loss-of-function (*spt* mutant). (**i**) Simulation of *RPL* loss-of-function (*rpl* mutant).

A further validation analysis for our proposed A. thaliana DZ model consisted of simulating constitutive loss- (LOF) and gain-of-function (GOF) mutations to compare the recovered attractors with the experimentally reported fruit phenotypes and their corresponding expression profiles, when data were available. LOF and GOF mutant simulations were performed by fixing the expression level of the corresponding node to 0 or 1, respectively. In most cases, the predicted configurations showed a high degree of coherence with the experimental data, as we show in Figure 7, with representative examples of simulations for experimentally well-supported mutant phenotypes reported in the literature, such as those in FUL, RPL, SHP, IND, ALC, and our newly proposed NTT node.

According to the literature, the carpel valves of *ful* mutants turn into valve margin-like tissue, with ectopic lignification and separation layer-like cell types. As a result, these cells fail to expand, and no stomatal precursor cells are present. However, the replum is not affected and continues to grow, adopting a zigzag morphology. The boundaries among the valves and the replum are indistinguishable [52]. Conversely, as a consequence of constitutive expression of *FUL*, the cells comprising the valve margin and outer replum of these indehiscent fruits convert to valve cells, and the dehiscence zone fails to differentiate [8]. In the null *ful* mutant, our model successfully recovered a three-attractor configuration with no valve (Figure 7a), and in the simulation of *FUL* constitutive activation, two converging valve-like configurations were obtained (Figure 7b).

Similarly, we found a perfect match with the predicted attractor corresponding to LOF RPL configuration (Figure 7i). In the *rpl* mutant, the replum region is replaced by a reduced number of narrow files of valve margin identity cells, leaving a nearly imperceptible separation domain between the fruit valves in the most severe *rpl* alleles [9].

We also recovered consistent configurations with both *ind* (Figure 7f) and *alc* (Figure 7g) knockouts, as well as for *NTT* and *SHP* overexpression lines (Figure 7d,e), exhibiting indehiscent fruits in all cases [5,6,44,49]. In all these cases, the attractors recovered by the model were those expected, taking into account the phenotype of the mutants: the loss of SL in *alc* mutants, the full absence of SL and LL in *ind*, the *ful*-like phenotypes caused by *NTT* overexpression, or the valve-to-LL shift in *SHP* overexpression lines.

While significantly consistent with the experimental data and the proposed roles of the master regulators of DZ formation, the model is not completely accurate in its predictions and the simulations of some of the reported mutant phenotypes, likely due to the limitations of this discrete (1 or 0) Boolean formalism. For instance, *SHP* LOF mutant mature siliques show no apparently developmental alterations in mature *shp1 shp2* fruits, except for the lack of lignified and separation layer specification at the valve margin domain, thus failing to dehisce [5]. So, on this occasion, due to the previously mentioned methodological restrictions, we had to leave out the reported quantitative regulation on *IND* expression by ALC and SPT [24] to prevent IND activity in the predicted attractors for the double knockout *shp1 shp2* mutants (Figure 7c). Moreover, there is still an unknown *IND* activator since the expression of *IND* is detected throughout the *shp1 shp2 ful* valves [8]. Actually, this possibility is reinforced by ectopic *IND* expression in our simulations of LOF of *SPT* (Figure 7h) and *ALC* (Figure 7g). So, this is one of the case examples that point out the need for further experimental testing to unveil the whole set of nodes and interactions with evidence of the potential of Boolean modeling to address these complex GRNs.

## 3. Materials and Methods

### 3.1. Data Integration and DZ GRN-Building

The inherent complexity of biological systems makes Boolean formalism a highly efficient approach to simplify the interplay between molecular regulators and changes in gene expression levels [53,54,55,56,57]. In our DZ GRN-building, we implemented qualitative Boolean modeling to depict the dynamics of genetic interactions determining the cellular pattern of the Arabidopsis fruit medio-lateral axis. With this aim, a comprehensive compilation of publicly available experimental data was performed for GRN topology architecture inferred from both the reported function of fruit dehiscence regulators and the demonstrated interactions between them (Figure 3a). In this network, each node represents a dehiscence or fruit developmental regulator, while the corresponding connections define the functional interactions between them.

Next, once the network topology outline was obtained, an initial set of logical rules was proposed to determine the joint action of these regulators on their targets, with the subsequent modification of either the states of their nodes or expression profiles along each time step. Thereby, the state of each transcriptional regulator was updated according to the function *x*_*i*_ (*t* + 1) = *f*_*i*_ (*x*_*i*1_ (*t*),…, *x*_*i*m_ (*t*)), where *x*_*i*_ represents the state of regulator *i* at time t + 1, which is given as a function of the state of its *m* regulators at time *t*. Given the nature of the model, each node of the network can display two possible states, 0 or 1, representing transcriptional inactivation or activation, respectively, *x*_*i*_ ∈ {0,1}. *S*(*t*) is the set of states of the *n* nodes that form the network or network configuration at time *t*, wherein the set of Boolean functions {*f*_1_,…,*f*_n_} determines the transition *S*(*t*)→*S*(*t* + 1). In this model, all nodes were updated simultaneously at each time step, i.e., synchronously. For a detailed description of the methodology of Boolean models, we recommend reviewing Saadatpour and Albert (2013), or Schwab et al (2020) [58,59].

Following this Boolean network approach, a preliminary dynamic fruit dehiscence network model (Figure 3a,b) was proposed with the help of the *BoolNet* library functions in *R* [27,60], which examined the network dynamics and obtained the attractors characterizing the expression profiles of the emerging cell types. Next, the exhaustive exploration of the set of logical rules proposed from experimental evidence was performed using the *Griffin* tool, using the *graph-R* notation described by Muñoz and colleagues [28]. Interactions with robustly described experimental evidence were established as MPU and MNU interactions (mandatory, positive, unambiguous, and mandatories, negative, unambiguous, respectively) as appropriate, while those interactions with putative involvement in fruit dehiscence were established as OPPA and ONPA interactions (optional, positive, possibly ambiguous, and optional, negative, possibly ambiguous, respectively). Figure 3 shows the results obtained with the *Griffin* tool from the initial network.

### 3.2. Identification and Analysis of Additional Interactions from TF2Network and PlantRegMap Databases

The output generated by Griffin demonstrated the need to include additional information since the set of regulators and/or interactions considered so far were not sufficient to propose optimally simulation-based interactions to support the so-far-described GRN explaining the emergence of the four different DZ domains/configurations. So, we searched for extra data in the specific TF2Network and PlantRegMap regulomics databases [37,38,61,62].

In the TF2Network platform, FUL, ARF6, ARF8, miR172, JAG, FIL, YAB3, AS1, AS2, AP2, SHP1, SHP2, IND, ALC, RPL, BP, and NTT were used as the set of input genes sharing a common Gene Ontology (GO) term of interest. The results were filtered according to the highest score assigned to the experimental validation of protein–DNA interactions. All experimental and hypothetical protein–DNA interactions, as well as PPIs, were downloaded. Simultaneously, the NETWORK tool included in the PlantRegMap repository was used to search for interactions between the same set of genes used as input in TF2Network. All exploration methods available in TF2Network were used, and experimental evidence was sought through Chip-Seq, literature mining, and the mapping of position weight matrix (PWMs), although it was only possible to find interactions through the latter two methods. Interactions specifically described in roots, root hair, and seedlings were eliminated. Finally, exploiting the additional information obtained, our whole pipeline was repeated to understand the dynamics of the newly generated regulation network and simulate the emerging attractors.

### 3.3. GRN Validation and Robustness Analysis

The ultimate DZ GRN was validated by constitutive expression (GOF) and LOF gene simulations for later comparison of these recovered attractors with the corresponding experimentally reported phenotypes of the simulated mutants. For this validation, the value of the node(s) of interest was set to “1” or “0” for gain- or LOF, respectively, as well as omitting the logical rules determining the transition state of the nodes.

The robustness of this novel network was comparatively evaluated with 1000 random networks with similar topological characteristics by simulating perturbations in 10% of the total logical functions or in 10% of the transition states, both for the DZ GRN and for the 1000 random copies. To analyze the dynamics of the mutant networks and perturbations to Boolean functions or transition states, functions from the BoolNet library in R [27,60] were also employed.

To discard the artefactual periodic behavior of some cyclic attractors obtained from the simulation of some mutants, these attractors were further evaluated following the methodology to generate continuous versions of Boolean models described in Mendoza or Méndez et al. [63,64]. The initial parameters, rates of change, as well as the corresponding steady states of this model can be consulted in the Supplementary File S3.

### 3.4. Bimolecular Fluorescence Complementation (BiFC)

Open reading the frames, full-length *NTT* and FIL CDS were cloned into vectors pYFPN43 and pYFPC43 (http://www.ibmcp.upv.es/PlantStressProteostasisLabVectors (accessed on 9 October 2024)).

Overnight grown cultures of A. tumefaciens C58/pMP90 of about 2.0 OD_600_ units were collected and resuspended in a similar volume of infiltration buffer (MgCl_2_ 10 mM, MES 10 mM pH 5.6, acetosyringone 200 µM) and incubated in a rocking platform at room temperature for 3 h at 50 rpm. To suppress gene silencing, A. tumefaciens cells expressing the p19 protein of the tomato bushy stunt virus were used in the co-infiltration procedure. A mixture of Agrobacterium strains containing the fluorescent translational fusion constructs pYFPN43, pYFPC43, and the p19 plasmid (1:1:0.5) was prepared for co-infiltration into the abaxial face of *N. benthamiana* leaves with a needleless syringe. The epidermal cell layers of at least three transformed leaves were assayed for fluorescence under a confocal microscope three days after infiltration. The experiments were repeated at least three times for every combination.

The samples were observed by confocal microscopy (Leica TCSSL, Wetzlar, Germany). Negative controls for interactions of NTT and FIL are shown in Supplemental File S4.

### 3.5. Luciferase Assays

To generate LUC fusions for transient expression assays in *Nicotiana benthamiana* leaves, promoter regions of *FUL* (2.3 kb, -2371 to ATG) and *SHP2* (2 kb, -2063 to ATG) were amplified from Arabidopsis Col-0 genomic DNA with primer pairs and cloned into pGREEN_LUC [65].

The effector constructs were generated by amplifying the corresponding ORFs of *NTT* and *FIL* from cDNA of Arabidopsis Col-0 inflorescences that were subsequently cloned into PCR8/GW/TOPO (Invitrogen, Carlsbad, CA, USA) and then transferred by Gateway reactions into the pMCD32 destination vector. The transient expression assays were performed by Agrobacterium-infiltrated transient transformation of *N. benthamiana*. Briefly, *N. benthamiana* plants were grown until they were about 5 cm in height. Approximately 300 μL of Agrobacterium containing the reporter or/and effector plasmids was infiltrated into a young leaf at three points. Firefly luciferase and Renilla luciferase were assayed 3 days after infiltration using the Dual-Luciferase Reporter Assay System (Promega, Madison, WI, USA). Data were represented as the ratio of LUC/REN and normalized to the values of the reporter construct when infiltrated alone. At least three plants at the same developmental stage were used for each treatment, and the experiment was repeated three times. A Student’s t-test was used to determine the significance of relative LUC activity differences.

## 4. Conclusions

Our work is a summary of the insufficient experimental data used until now, conducted to propose a genetic model for the differentiation of the DZ in Arabidopsis, which is not complete and lacks essential components. Hence, we were able to propose a set of hypothetical rules and the incorporation of the additional NTT factor as a putative crucial component of the GRN directing the differentiation of the Arabidopsis DZ to successfully recover the observed developmental outputs. The model proposed here still requires extensive experimental validation that should be undertaken in the future to confirm or reformulate our proposal. In any case, it provides a new framework to feed further work in the field and to identify new avenues for biotechnological manipulation of fruit characters in crop species.

## Figures and Tables

**Table 1:** The compiled set of nodes and its experimental interactions and references. The asterisks indicate those considered in the first version of the model, while the inclusion of NTT and the remaining interactions were considered in our final proposed model.

			Experimentally Well-Supported Interactions	
Regulator		Target	Description	Refs.
* IND	→	ALC	*ALC* transcripts are not detected at the valve margin of *ind* mutants.	[5,24]
SHP1/2	→	ALC	*ALC* transcripts are not detected at the valve margin of *shp1 shp2* fruits.	[6,7]
* miR172	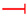	AP2	Proper valve growth depends on the post-transcriptional limitation of AP2 activity by miR172 repression. Fruit phenotypes reminiscent of *ful* mutants are observed in transgenic plants expressing a miR172-resistant version of *AP2* (*FUL>>rAP2*). Additionally, reduced activity of mature miR172 results in an overall reduction in fruit size.	[19]
* AP2	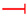	ARF6/8	AP2 directly promotes the expression of *AGL15*, which in turn acts as an auxin signaling repressor through the negative regulation of *ARF6* and *ARF8*.	[29,30]
* AP2	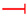	AS1/2	While AP2 and AS1/2 are both negative regulators of *BP* and *RPL*, AP2 appears to maintain AS1/2 activity confined to the valves. The direct binding of the AP2 protein 1.2 kb upstream from the *AS1* ATG transcription start codon and the synergistic effect of *ap2* and *as1* mutations on replum size are consistent with this proposed interaction.	[18,29]
AP2	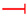	BP	*ap2* mutations result in an enlarged replum together with a prominent increase in the expression levels of *RPL::GUS* and *BP::GUS* reporters and the expansion of their expression domains. This direct or indirect repression is also supported by significantly higher levels of *RPL* and *BP* transcripts in *ap2* carpels than in the wild-type background. The major role of AP2 as a suppressor of replum overgrowth is further confirmed as *rpl* and *bp* mutations mitigate *ap2* replum defects.	[18]
* AS1/2	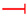	BP	The ectopic expression of *BP* is detected in lateral regions of *as1* carpels, together with fruit defects resembling 35S::BP plants. Furthermore, the almost complete restoration of wild-type replum and valve size is evident in *as1 bp* fruits.	[12]
* SHP1/2	→	IND	*IND* expression is missing in *shp1 shp2* indehiscent fruits, which display remarkable phenotypic similarities to *ind* mutant alleles.	[5,6,8]
* BP	→	RPL	BP positively regulates the expression of the *RPL* promoter. This activation is confirmed by qRT-PCR, which shows an increase in *RPL* transcripts in *35S::BP* plants compared with the No-0 background.	[12]
* JAG/FIL/YAB3	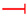	BP	The phenotypic similarity between loss of function *jag fil* mutants and 35S::BP fruits, as well as the partial suppression of the *jag fil* phenotype in a *bp* background, supports the negative regulation of *BP* by JAG/FIL/YAB3 lateral factors. In addition, an increased and expanded BP::GUS signal, along with higher expression levels of *BP* in pistils with compromised JAG/FIL activity confirmed this previous evidence.	[15]
NTT	→	BP	The overexpression of *NTT* ectopically activates *BP* expression.	[21]
* BP	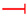	JAG/FIL/YAB3	Decreased *JAG* and *FIL* expression is detected when *BP* is ectopically expressed. This negative regulation of BP on JAG/FIL activity is further confirmed by qRT-PCR mRNA quantification.	[15]
FUL	→	FUL	The *FUL* locus itself was significantly enriched in a FUL ChIP-Seq experiment aiming at identifying the direct targets of this transcription factor.	[31]
ALC	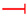	IND	In *alc* mutants, *IND* expression is increased.	[24]
* AP2	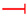	IND	The role of AP2 as an *IND* repressor is suggested by both increased levels and wider domains of *IND::GUS* expression in *ap2* mutants. This repression is not mediated by SHP, since in *ap2 shp1 shp2* mutants *IND* expression is detected at the valve margin, as opposed to its total absence in *shp1 shp2* mutants.	[18]
* FUL	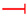	IND	*IND* is ectopically expressed in *ful* mutant fruits and absent in 35S::FUL fruits.	[8]
* FUL (+ARF6/8)	→	miR172	The physical interaction between FUL and ARF6/8 in plants promotes *miR172C* expression in the valves, most probably by directly binding to CArG and AuxREs motifs in the *miR172C* promoter. Decreased relative transcript levels of *miR172C* as well as the dramatic reduction in *miR172C::GUS* expression in *ful* and *arf6/8* mutant combinations confirms the role of both FUL and ARF6/8 as positive regulators of miR172C activity.	[19]
* AP2	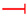	RPL	*ap2* mutations result in an enlarged replum, together with a prominent increase in the expression levels of *RPL::GUS* and *BP::GUS* reporters and the expansion of their expression domains. This direct or indirect repression is also supported by significantly higher levels of *RPL* and *BP* transcripts in *ap2* carpels than in the wild-type background. The major role of AP2 as a suppressor of replum overgrowth is further confirmed as *rpl* and *bp* mutations mitigate *ap2* replum defects.	[18]
* JAG/FIL/YAB3	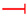	RPL	*RPL* expression levels are considerably increased in *fil yab3 bp* and *fil jag bp* pistils with respect to the wild-type and *bp* genetic backgrounds, despite the low impact of defective BP activity on RPL function, thus revealing repressive JAG/FIL/YAB3 activity on this replum gene.	[15]
* AP2	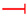	SHP1/2	Consistent with the increased size of the lignification layer in the valve margin of *ap2* mutants, the *SHP2::GUS* expression domain broadens and the higher expression levels in *ap2* mutants suggest that AP2 acts as a negative regulator of SHP activity.	[18]
* FUL	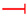	SHP1/2	*ful* mutants show ectopic *SHP1* and *SHP2* expression throughout the valves, contrary to the *SHP* down-regulation detected in *35S::FUL* lines. ChIP-Seq experiments demonstrate the repression of *SHP2* by direct FUL binding to CArG boxes located within 1000 bp at the start of the gene.	[8,13,31]
* JAG/FIL/YAB3	→	SHP1/2	As it occurs with *FUL*, in *fil yab3* mutants, *SHP2* expression is lost during the early development stages. In a redundant manner, JAG, together with FIL and YAB3, promote *SHP* expression, which is further reduced in *jag fil yab3* mutants when compared to *fil yab3* backgrounds.	[13,14,15]
* FUL	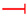	ALC	*ful* knock-out mutants display ectopic expression of the valve margin identity genes in the valves, conferring valve margin-like development, with the ectopic formation of lignified and separation layer-like cell types.	[8,13]
AP2	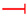	AP2	AP2 activity represses its own expression.	[32,33,34,35]
FUL	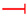	AP2	FUL directly and negatively regulates *AP2* expression in the shoot apical meristem.	[36,37,38]
AS1/2	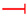	JAG/FIL/YAB3	Transcript levels of *FIL* and *YAB3* are reduced in *35S:AS2* shoots compared with those in the wild-type.	[37,38,39,40]
* JAG/FIL/YAB3	→	FUL	FIL and YAB3 promote *FUL* expression in the valves. In *fil yab3* mutants, *FUL* expression is absent from valves in both the apical and basal regions. In addition, *FUL* expression decreases in *jag* single mutants which partly resemble *ful* mutant phenotypes, and are enhanced in *jag fil* and *jag fil yab3* +/− fruits. These results suggest redundant JAG activity with FIL and YAB3 to promote *FUL* expression in the valves.	[13,14,15,41,42]
NTT	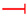	JAG/FIL/YAB3	RNA-Seq results show that *FIL* and *YAB3* genes are differentially expressed upon induction in NTT activity. When *NTT* expression is strongly induced, *FIL* expression levels are significantly repressed, most probably in an indirect manner, since their genomic regions were not identified as enriched in the corresponding ChIP-Seq experiment.	[43]
AP2	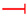	miR172	Multiple lines of evidence point to the direct negative regulation of *miR172* by AP2 binding. The ChIP-Seq results are in line with the similar pleiotropic phenotypes of *ap2* mutants, similar to those observed in *35S:miR172* overexpressors, together with a higher significant abundance of miR172 gel blots on inflorescence tissue from *ap2-2* mutants.	[29,37]
NTT	→	NTT	The autoactivation of *NTT* is confirmed both in yeast and in planta by Y1H and BiFC, respectively.	[44]
FUL	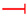	SPT	The repression of *SPT* expression by FUL activity is demonstrated by the ectopic expression of *pSPT-1262:GUS* throughout the *ful* mutant valves.	[10]
IND	→	SPT	IND directly activates *SPT* expression consistent with the overlapping expression patterns of *IND* and *SPT* in the valve margin. BiFC in *Nicotiana tabacum* cells confirms nuclear localization of IND protein and in vivo interaction with SPT.	[11,24,45]
SHP1/2	→	SPT	In *shp1-1 shp2-1* double mutants, *pSPT-1262:GUS* expression is weaker and more diffused compared with WT fruits, although it can be still detected. So, *SPT* activation is only partially dependent on SHP.	[10,45]
			**Experimentally Suggested Interactions**	
* RPL	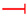	FIL	*rpl* mutation results in the expansion of *FIL* expression into the replum and its conversion into valve margin. Both impaired JAG or FIL activity in an *rpl* mutant background rescues replum development.	[13]
* AS1/2	→	FUL	The GUS expression pattern driven by the *FUL* promoter in *as1-104* mutants shows a severely decreased signal, as expected for these bumpy fruits.	[12]
BP	→	BP	*BP:GUS* expression (and perhaps other KNOX genes) may be upregulated in inflorescence areas where *BP* expression overlaps.	[37,38,46,47]
SHP1/2	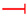	AS1/2	Repression is suggested by the position weight matrix (PWM).	[37,38]
			**Reported Protein–Protein (PPIs) Interactions**	
IND	—	SHP1/2	PPI.	[37,38]
BP	—	AP2	PPI.	[46]
FIL	—	AP2	PPI.	[46]
NTT	—	AS1/2	PPI.	[47]
RPL	—	BP	PPI.	[37,48]
SHP1/2	—	BP	PPI.	[48]
AP2	—	FIL	PPI.	[48]
SHP1/2	—	FUL	PPI.	[37]
RPL	—	IND	PPI.	[38]
JAG/YAB3	—	JAG/YAB3	PPI.	[37]
NTT	—	FUL	PPI.	[21,44,48,49]
NTT	—	RPL	PPI.	[49]
NTT	—	SHP1/2	PPI.	[48,49]
BP	—	SHP1/2	PPI.	[46]

## Data Availability

All data are contained within the article.

## Supplementary Materials

The following supporting information can be downloaded at https://www.mdpi.com/article/10.3390/plants13202927/s1, Table S1: Logical rules derived from the starting set of 11 nodes and 22 experimentally validated interactions that only recovered three stable attractors which lack proper differentiation of lignification and separation layers present in the Arabidopsis dehiscent fruit.; Table S2: Logical rules including both novel proposed regulatory interactions and the NTT node are sufficient to recover the expected four attractors corresponding to a dehiscent fruit.; Supplementary File S3: Continuous version of *A. thaliana* DZ GRN model; Supplementary File S4: Controls for BiFC experiment.
