# Supplementary material for: A Model for the Gene Regulatory Network Along the Arabidopsis Fruit Medio-Lateral Axis: Rewiring the Pod Shatter Process
Source: Plants (Basel). 2024 Oct 18;13(20):2927. doi: 10.3390/plants13202927 (PMC11511003; doi:10.3390/plants13202927)
Supplement: Supplementary file 1 [file plants-13-02927-s001.zip › Supp_File_S3.pdf]

Supplementary File 3:
Continuous version of *A. thaliana* DZ GRN model

The continuous model of the DZ GRN was implemented for a deeper examination of the periodic expression obtained with the JAG/YAB3 KO and miR172 OE mutants. The valve, replum, LL and SL attractors in the WT background were also assessed, confirming the constant expression for all regulators as in the Boolean model (not shown). This model was built following the methodology described in [1, 2].

miR172 OE

Parameters

$h = 50$
$\gamma = 1$

$$\omega_{FUL} = min(max(JAG/YAB3,\ FIL),\ max(min(FUL,\ 1-NTT),\ AS1/2))$$
$$\omega_{AS1/2} = min(1-AP2,\ 1-SHP1/2)$$
$$\omega_{ARF6/8} = 1-AP2$$
$$\omega_{miR172} = 1$$
$$\omega_{JAG/YAB3} = min(max(SHP1/2,\ 1-BP),\ 1-RPL,\ max(JAG/YAB3,\ 1-min(AS1/2,\ 1-NTT)))$$
$$\omega_{FIL} = min(1-RPL,\ max(1-BP,\ SHP1/2),\ 1-min(AS1/2,\ 1-NTT))$$
$$\omega_{AP2} = min(max(1-miR172,\ 1-AP2),\ max(1-FUL,\ NTT))$$
$$\omega_{SHP1/2} = min(max(max(SHP1/2,\ 1-BP),\ JAG/YAB3,\ FIL,\ 1-AP2),\ max(1-FUL,\ NTT),\ 1-RPL)$$
$$\omega_{IND} = min(SHP1/2,\ max(max(1-FUL,\ NTT),\ min(1-BP,\ 1-RPL,\ 1-min(AP2,\ 1-FIL))))$$
$$\omega_{SPT} = min(min(IND,\ SHP1/2),\ max(1-FUL,\ NTT))$$
$$\omega_{ALC} = min(min(SHP1/2,\ NTT,\ IND,\ SPT),\ 1-FUL,\ 1-RPL)$$
$$\omega_{NTT} = min(NTT,\ max(FIL,\ SHP1/2),\ 1-FUL,\ 1-AS1/2,\ 1-RPL)$$
$$\omega_{RPL} = min(max(min(BP,\ 1-SHP1/2),\ RPL,\ 1-AP2),\ 1-JAG/YAB3,\ 1-FIL,\ 1-FUL)$$
$$\omega_{BP} = min(max(RPL,\ NTT,\ min(BP,\ 1-max(AP2,\ SHP1/2))),\ 1-JAG/YAB3,\ 1-FIL,\ 1-AS1/2)$$

Rates of change

$$\frac{dFUL}{dt} = \frac{-e^{0.5h} + e^{-h(\omega_{FUL}-0.5)}}{(1-e^{0.5h})(1+e^{-h(\omega_{FUL}-0.5)})} - \gamma FUL$$
$$\frac{dAS1/2}{dt} = \frac{-e^{0.5h} + e^{-h(\omega_{AS1/2}-0.5)}}{(1-e^{0.5h})(1+e^{-h(\omega_{AS1/2}-0.5)})} - \gamma AS1/2$$

$$\frac{dARF6/8}{dt} = \frac{-e^{0.5h} + e^{-h(\omega_{ARF6/8}-0.5)}}{(1-e^{0.5h})(1+e^{-h(\omega_{ARF6/8}-0.5)})} - \gamma ARF6/8$$

$$\frac{dmiR172}{dt} = 0$$

$$\frac{dJAG/YAB3}{dt} = \frac{-e^{0.5h} + e^{-h(\omega_{JAG/YAB3}-0.5)}}{(1-e^{0.5h})(1+e^{-h(\omega_{JAG/YAB3}-0.5)})} - \gamma JAG/YAB3$$

$$\frac{dFIL}{dt} = \frac{-e^{0.5h} + e^{-h(\omega_{FIL}-0.5)}}{(1-e^{0.5h})(1+e^{-h(\omega_{FIL}-0.5)})} - \gamma FIL$$

$$\frac{dAP2}{dt} = \frac{-e^{0.5h} + e^{-h(\omega_{AP2}-0.5)}}{(1-e^{0.5h})(1+e^{-h(\omega_{AP2}-0.5)})} - \gamma AP2$$

$$\frac{dSHP1/2}{dt} = \frac{-e^{0.5h} + e^{-h(\omega_{SHP1/2}-0.5)}}{(1-e^{0.5h})(1+e^{-h(\omega_{SHP1/2}-0.5)})} - \gamma SHP1/2$$

$$\frac{dIND}{dt} = \frac{-e^{0.5h} + e^{-h(\omega_{IND}-0.5)}}{(1-e^{0.5h})(1+e^{-h(\omega_{IND}-0.5)})} - \gamma IND$$

$$\frac{dSPT}{dt} = \frac{-e^{0.5h} + e^{-h(\omega_{SPT}-0.5)}}{(1-e^{0.5h})(1+e^{-h(\omega_{SPT}-0.5)})} - \gamma SPT$$

$$\frac{dALC}{dt} = \frac{-e^{0.5h} + e^{-h(\omega_{ALC}-0.5)}}{(1-e^{0.5h})(1+e^{-h(\omega_{ALC}-0.5)})} - \gamma ALC$$

$$\frac{dNTT}{dt} = \frac{-e^{0.5h} + e^{-h(\omega_{NTT}-0.5)}}{(1-e^{0.5h})(1+e^{-h(\omega_{NTT}-0.5)})} - \gamma NTT$$

$$\frac{dRPL}{dt} = \frac{-e^{0.5h} + e^{-h(\omega_{RPL}-0.5)}}{(1-e^{0.5h})(1+e^{-h(\omega_{RPL}-0.5)})} - \gamma RPL$$

$$\frac{dBP}{dt} = \frac{-e^{0.5h} + e^{-h(\omega_{BP}-0.5)}}{(1-e^{0.5h})(1+e^{-h(\omega_{BP}-0.5)})} - \gamma BP$$

Results for the initial condition: $FUL = 0,\ AS1/2 = 0,\ ARF6/8 = 0,\ miR172 = 1,\ JAG/YAB3 = 0,\ FIL = 0,\ AP2 = 0,\ SHP1/2 = 0,\ IND = 0,\ SPT = 0,\ ALC = 0,\ NTT = 0,\ RPL = 1,\ BP = 0$

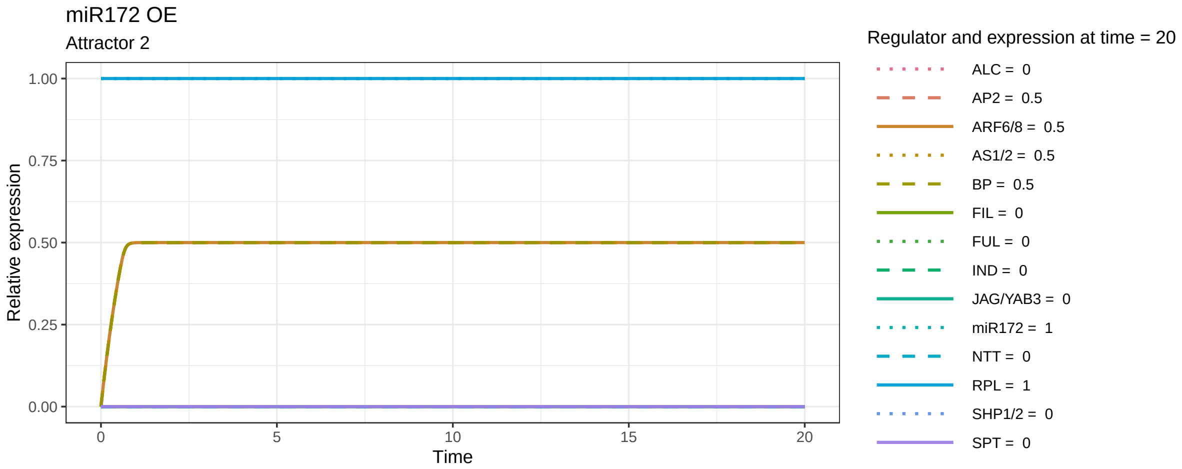

Figure 1: Attractor obtained with the continuous form of the DZ GRN model with the miR172 OE background. The initial condition corresponds to one configuration of the cyclic attractor 2 obtained in the Boolean model. In contrast to the Boolean model, after $time = 3$, all node states remain constant, dismissing a possible cyclic behaviour of this dynamic GRN model.

Results for the initial condition: $FUL = 0,\ AS1/2 = 0,\ ARF6/8 = 0,\ miR172 = 1,\ JAG/YAB3 = 1,$ $FIL = 1,\ AP2 = 0,\ SHP1/2 = 1,\ IND = 1,\ SPT = 1,\ ALC = 0,\ NTT = 0,\ RPL = 0,\ BP = 0$

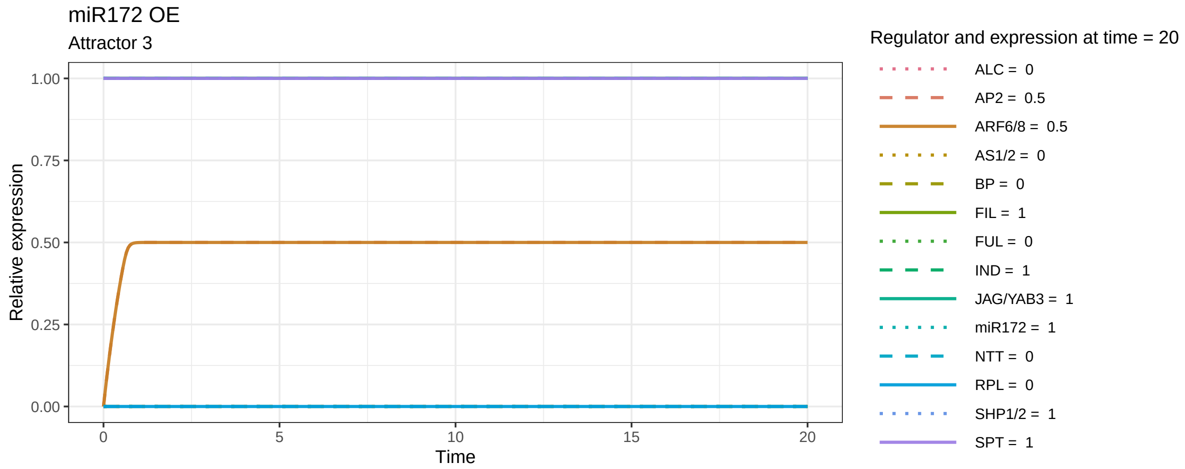

Figure 2: Attractor obtained with the continuous form of the DZ GRN model with the miR172 OE background. The initial condition corresponds to one configuration of the cyclic attractor 3 obtained in the Boolean model. After $time = 3$, all node states remain constant.

Results for the initial condition: $FUL = 0,\ AS1/2 = 0,\ ARF6/8 = 0,\ miR172 = 1,\ JAG/YAB3 = 1,$ $FIL = 1,\ AP2 = 0,\ SHP1/2 = 1,\ IND = 1,\ SPT = 1,\ ALC = 1,\ NTT = 1,\ RPL = 0,\ BP = 0$

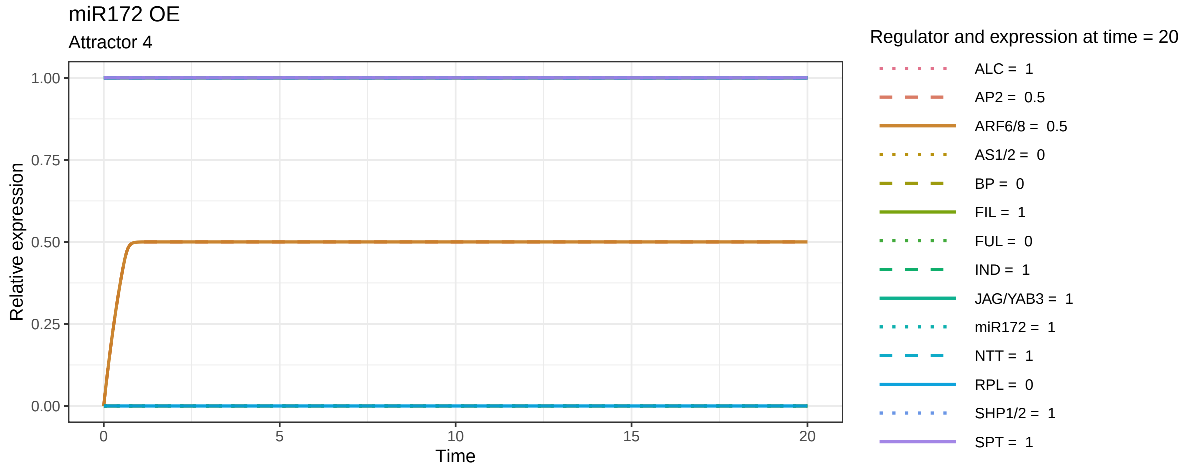

Figure 3: Attractor obtained with the continuous form of the DZ GRN model with the miR172 OE background. The initial condition corresponds to one configuration of the cyclic attractor 4 obtained in the Boolean model. Once again, after $time = 3$, all node states remain constant.

JAG/YAB3 KO

Parameters

$h = 50$
$\gamma = 1$

$$\begin{aligned}
\omega_{FUL} &= min(max(JAG/YAB3,\ FIL),\ max(min(FUL,\ 1-NTT),\ AS1/2)) \\
\omega_{AS1/2} &= min(1-AP2,\ 1-SHP1/2) \\
\omega_{ARF6/8} &= 1-AP2 \\
\omega_{miR172} &= min(min(ARF6/8,\ min(FUL,\ 1-NTT)),\ 1-AP2) \\
\omega_{JAG/YAB3} &= 0 \\
\omega_{FIL} &= min(1-RPL,\ max(1-BP,\ SHP1/2),\ 1-min(AS1/2,\ 1-NTT)) \\
\omega_{AP2} &= min(max(1-miR172,\ 1-AP2),\ max(1-FUL,\ NTT)) \\
\omega_{SHP1/2} &= min(max(max(SHP1/2,\ 1-BP),\ JAG/YAB3,\ FIL,\ 1-AP2),\ max(1-FUL,\ NTT),\ 1-RPL) \\
\omega_{IND} &= min(SHP1/2,\ max(max(1-FUL,\ NTT),\ min(1-BP,\ 1-RPL,\ 1-min(AP2,\ 1-FIL)))) \\
\omega_{SPT} &= min(min(IND,\ SHP1/2),\ max(1-FUL,\ NTT)) \\
\omega_{ALC} &= min(min(SHP1/2,\ NTT,\ IND,\ SPT),\ 1-FUL,\ 1-RPL) \\
\omega_{NTT} &= min(NTT,\ max(FIL,\ SHP1/2),\ 1-FUL,\ 1-AS1/2,\ 1-RPL) \\
\omega_{RPL} &= min(max(min(BP,\ 1-SHP1/2),\ RPL,\ 1-AP2),\ 1-JAG/YAB3,\ 1-FIL,\ 1-FUL) \\
\omega_{BP} &= min(max(RPL,\ NTT,\ min(BP,\ 1-max(AP2,\ SHP1/2))),\ 1-JAG/YAB3,\ 1-FIL,\ 1-AS1/2)
\end{aligned}$$

Rates of change

$$\begin{aligned}
\frac{dFUL}{dt} &= \frac{-e^{0.5h} + e^{-h(\omega_{FUL}-0.5)}}{(1-e^{0.5h})(1+e^{-h(\omega_{FUL}-0.5)})} - \gamma FUL \\
\frac{dAS1/2}{dt} &= \frac{-e^{0.5h} + e^{-h(\omega_{AS1/2}-0.5)}}{(1-e^{0.5h})(1+e^{-h(\omega_{AS1/2}-0.5)})} - \gamma AS1/2 \\
\frac{dARF6/8}{dt} &= \frac{-e^{0.5h} + e^{-h(\omega_{ARF6/8}-0.5)}}{(1-e^{0.5h})(1+e^{-h(\omega_{ARF6/8}-0.5)})} - \gamma ARF6/8 \\
\frac{dmiR172}{dt} &= \frac{-e^{0.5h} + e^{-h(\omega_{miR172}-0.5)}}{(1-e^{0.5h})(1+e^{-h(\omega_{miR172}-0.5)})} - \gamma miR172 \\
\frac{dJAG/YAB3}{dt} &= 0 \\
\frac{dFIL}{dt} &= \frac{-e^{0.5h} + e^{-h(\omega_{FIL}-0.5)}}{(1-e^{0.5h})(1+e^{-h(\omega_{FIL}-0.5)})} - \gamma FIL \\
\frac{dAP2}{dt} &= \frac{-e^{0.5h} + e^{-h(\omega_{AP2}-0.5)}}{(1-e^{0.5h})(1+e^{-h(\omega_{AP2}-0.5)})} - \gamma AP2 \\
\frac{dSHP1/2}{dt} &= \frac{-e^{0.5h} + e^{-h(\omega_{SHP1/2}-0.5)}}{(1-e^{0.5h})(1+e^{-h(\omega_{SHP1/2}-0.5)})} - \gamma SHP1/2 \\
\frac{dIND}{dt} &= \frac{-e^{0.5h} + e^{-h(\omega_{IND}-0.5)}}{(1-e^{0.5h})(1+e^{-h(\omega_{IND}-0.5)})} - \gamma IND
\end{aligned}$$

$$\frac{dSPT}{dt} = \frac{-e^{0.5h} + e^{-h(\omega_{SPT}-0.5)}}{(1-e^{0.5h})(1+e^{-h(\omega_{SPT}-0.5)})} - \gamma SPT$$

$$\frac{dALC}{dt} = \frac{-e^{0.5h} + e^{-h(\omega_{ALC}-0.5)}}{(1-e^{0.5h})(1+e^{-h(\omega_{ALC}-0.5)})} - \gamma ALC$$

$$\frac{dNTT}{dt} = \frac{-e^{0.5h} + e^{-h(\omega_{NTT}-0.5)}}{(1-e^{0.5h})(1+e^{-h(\omega_{NTT}-0.5)})} - \gamma NTT$$

$$\frac{dRPL}{dt} = \frac{-e^{0.5h} + e^{-h(\omega_{RPL}-0.5)}}{(1-e^{0.5h})(1+e^{-h(\omega_{RPL}-0.5)})} - \gamma RPL$$

$$\frac{dBP}{dt} = \frac{-e^{0.5h} + e^{-h(\omega_{BP}-0.5)}}{(1-e^{0.5h})(1+e^{-h(\omega_{BP}-0.5)})} - \gamma BP$$

Results for the initial condition:
$FUL = 0,\ AS1/2 = 0,\ ARF6/8 = 0,\ miR172 = 1,\ JAG/YAB3 = 0,$
$FIL = 1,\ AP2 = 0,\ SHP1/2 = 0,\ IND = 0,\ SPT = 0,\ ALC = 0,\ NTT = 0,\ RPL = 0,\ BP = 0$

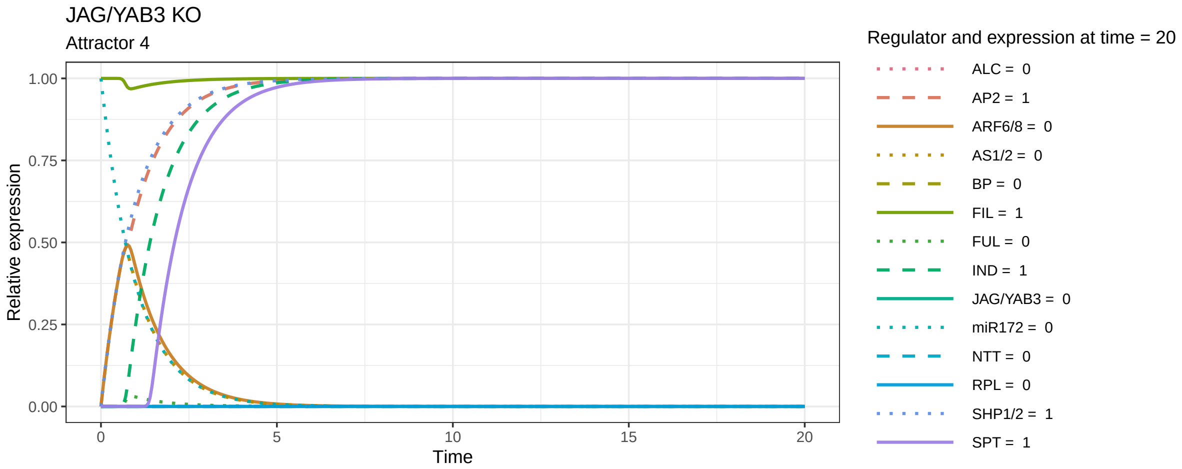

Figure 4: Attractor obtained with the continuous form of the DZ GRN model in the JAG/YAB3 KO background. The initial condition corresponds to one configuration of the cyclic attractor 4 obtained in the Boolean model. After $time = 7$, all node states remain constant, discarding other possible cyclic behaviour of this dynamic GRN model.

References

1. Mendoza, L. & Xenarios, I. A method for the generation of standardized qualitative dynamical systems of regulatory networks. en. *Theor. Biol. Med. Model.* **3,** 13 (Mar. 2006).

2. Méndez, A., Ramírez, C., Martínez, M. P. & Mendoza, L. The SQUAD method for the qualitative modeling of regulatory networks. en. *Methods Mol. Biol.* **1819,** 197–214 (2018).
