# Supplementary material for: A Model for the Gene Regulatory Network Along the Arabidopsis Fruit Medio-Lateral Axis: Rewiring the Pod Shatter Process
Source: Plants (Basel). 2024 Oct 18;13(20):2927. doi: 10.3390/plants13202927 (PMC11511003; doi:10.3390/plants13202927)
Supplement: Supplementary file 1 [file plants-13-02927-s001.zip › Supplementary File S4.pdf]

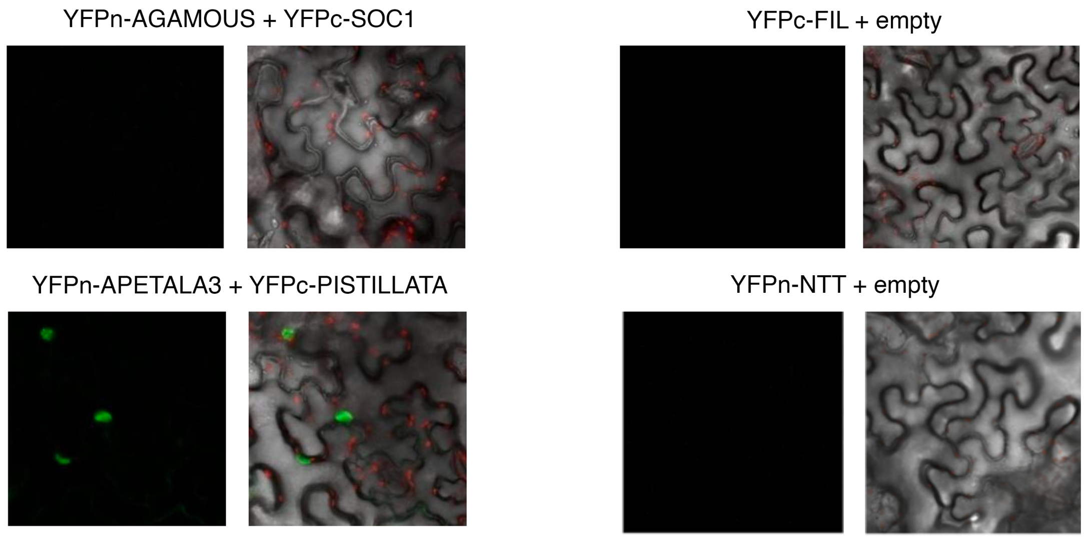

Supplementary File S4: Controls for BiFC experiment. For negative control The YFPc-FIL and YFPn-NTT constructs were co-infiltrated with the empty complementary vector (YFPn and YFPc respectively). AGAMOUS and SOC1 are unrelated MADS-box transcription factors known to not intercat that were used as an additional negative control. In all cases, no reconstituted fluorescence was observed.
Positive controls for the well-known interactions of the MADS box factors PISTILLATA with APETALA3 are also provided
