# Supplementary material for: A Model for the Gene Regulatory Network Along the Arabidopsis Fruit Medio-Lateral Axis: Rewiring the Pod Shatter Process
Source: Plants (Basel). 2024 Oct 18;13(20):2927. doi: 10.3390/plants13202927 (PMC11511003; doi:10.3390/plants13202927)
Supplement: Supplementary file 1 [file plants-13-02927-s001.zip › Table_S1.pdf]

<table>
<tr><th>Target</th><th>Rule</th></tr>
<tr><td>FUL</td><td>JAG/FIL/YAB3 & AS1/2</td></tr>
<tr><td>ARF6/8</td><td>!AP2</td></tr>
<tr><td>miR172</td><td>FUL & ARF6/8</td></tr>
<tr><td>JAG/FIL/YAB3</td><td>! BP I ! RPL</td></tr>
<tr><td>AS1/2</td><td>! AP2</td></tr>
<tr><td>AP2</td><td>! miR172</td></tr>
<tr><td>SHP1/2</td><td>JAG/FIL/YAB3 & (! AP2 | ! FUL)</td></tr>
<tr><td>IND</td><td>(SHP1/2 | ! AP2) & ! FUL</td></tr>
<tr><td>ALC</td><td>IND & ! FUL</td></tr>
<tr><td>RPL</td><td>! JAG/FIL/YAB3 & BP & ! AP2</td></tr>
<tr><td>BP</td><td>! JAG/FIL/YAB3 & ! AS1/2</td></tr>
</table>

Table S1. Logical rules derived from the starting set of 11 nodes and 22 experimentally validated interactions that only recovered three stable attractors which lack of proper differentiation of lignification and separation layers present in the Arabidopsis dehiscent fruit.
