# Supplementary material for: A Model for the Gene Regulatory Network Along the Arabidopsis Fruit Medio-Lateral Axis: Rewiring the Pod Shatter Process
Source: Plants (Basel). 2024 Oct 18;13(20):2927. doi: 10.3390/plants13202927 (PMC11511003; doi:10.3390/plants13202927)
Supplement: Supplementary file 1 [file plants-13-02927-s001.zip › Table_S2.pdf]

<table>
<tr><th>Target</th><th>Rule</th></tr>
<tr><td>FUL</td><td>(JAG/YAB3 | FIL) & ((FUL & ! NTT) | AS1/2)</td></tr>
<tr><td>AS1/2</td><td>! AP2 & ! SHP1/2</td></tr>
<tr><td>ARF6/8</td><td>! AP2</td></tr>
<tr><td>miR172</td><td>(ARF6/8 & (FUL & ! NTT)) & ! AP2</td></tr>
<tr><td>JAG/YAB3</td><td>(SHP1/2 | ! BP) & ! RPL & (JAG/YAB3 | ! (AS1/2 & ! NTT))</td></tr>
<tr><td>FIL</td><td>! RPL & (! BP I SHP1/2) & ! (AS1/2 & ! NTT)</td></tr>
<tr><td>AP2</td><td>(! miR172 I ! AP2) & (! FUL | NTT)</td></tr>
<tr><td>SHP1/2</td><td>((SHP1/2 | ! BP) | JAG/YAB3 | FIL | ! AP2) & (! FUL | NTT) & ! RPL</td></tr>
<tr><td>IND</td><td>SHP1/2 & ((! FUL | NTT) | (! BP & ! RPL & ! (AP2 & ! FIL)))</td></tr>
<tr><td>SPT</td><td>(IND & SHP1/2) & (! FUL | NTT)</td></tr>
<tr><td>ALC</td><td>(SHP1/2 & NTT & IND & SPT) & ! FUL & ! RPL</td></tr>
<tr><td>NTT</td><td>NTT & (FIL | SHP1/2) & ! FUL & ! AS1/2 & ! RPL)</td></tr>
<tr><td>RPL</td><td>((BP & ! SHP1/2) | RPL | ! AP2) & ! JAG/YAB3 & ! FIL & ! FUL</td></tr>
<tr><td>BP</td><td>(RPL | NTT | (BP & ! AP2 | SHP1/2)) & ! JAG/YAB3 & ! FIL & ! AS1/2</td></tr>
</table>

Table S2. Logical rules including both novel proposed regulatory interactions and the NTT node are sufficient to recover the expected four attractors corresponding to a dehiscent fruit.
